# Noninvasive Prenatal Diagnosis of Fetal Trisomy 21 by Allelic Ratio Analysis Using Targeted Massively Parallel Sequencing of Maternal Plasma DNA

**DOI:** 10.1371/journal.pone.0038154

**Published:** 2012-05-29

**Authors:** Gary J. W. Liao, K. C. Allen Chan, Peiyong Jiang, Hao Sun, Tak Y. Leung, Rossa W. K. Chiu, Y. M. Dennis Lo

**Affiliations:** 1 Centre for Research into Circulating Fetal Nucleic Acids, Li Ka Shing Institute of Health Sciences, The Chinese University of Hong Kong, Shatin, New Territories, Hong Kong, China; 2 Department of Chemical Pathology, The Chinese University of Hong Kong, Shatin, New Territories, Hong Kong, China; 3 Department of Obstetrics and Gynaecology, The Chinese University of Hong Kong, Shatin, New Territories, Hong Kong, China; VU University Medical Center, Netherlands

## Abstract

**Background:**

Plasma DNA obtained from a pregnant woman contains a mixture of maternal and fetal DNA. The fetal DNA proportion in maternal plasma is relatively consistent as determined using polymorphic genetic markers across different chromosomes in euploid pregnancies. For aneuploid pregnancies, the observed fetal DNA proportion measured using polymorphic genetic markers for the aneuploid chromosome would be perturbed. In this study, we investigated the feasibility of analyzing single nucleotide polymorphisms using targeted massively parallel sequencing to detect such perturbations in mothers carrying trisomy 21 fetuses.

**Methodology/Principal Findings:**

DNA was extracted from plasma samples collected from fourteen pregnant women carrying singleton fetuses. Hybridization-based targeted sequencing was used to enrich 2 906 single nucleotide polymorphism loci on chr7, chr13, chr18 and chr21. Plasma DNA libraries with and without target enrichment were analyzed by massively parallel sequencing. Genomic DNA samples of both the mother and fetus for each case were genotyped by single nucleotide polymorphism microarray analysis. For the targeted regions, the mean sequencing depth of the enriched samples was 225-fold higher than that of the non-enriched samples. From the targeted sequencing data, the ratio between fetus-specific and shared alleles increased by approximately 2-fold on chr21 in the paternally-derived trisomy 21 case. In comparison, the ratio is decreased by approximately 11% on chr21 in the maternally-derived trisomy 21 cases but with much overlap with the ratio of the euploid cases. Computer simulation revealed the relationship between the fetal DNA proportion, the number of informative alleles and the depth of sequencing.

**Conclusions/Significance:**

Targeted massively parallel sequencing of single nucleotide polymorphism loci in maternal plasma DNA is a potential approach for trisomy 21 detection. However, the method appears to be less robust than approaches using non-polymorphism-based counting of sequence tags in plasma.

## Introduction

Prenatal screening and diagnosis of fetal aneuploidies, such as trisomy 21 (T21), is an established part of modern obstetrics care. Conventional prenatal screening is built on parameters such as maternal age, sonographic and biochemical markers [1]. Since these parameters are mainly based on phenotypic features, which are essentially epiphenomena associated with the core molecular pathology, their diagnostic performance is usually suboptimal. Pregnancies stratified as high risk by the above screening approaches require further investigation of fetal genetic materials obtained via invasive procedures, such as chorionic villus sampling (CVS) and amniocentesis. These latter procedures carry small, but definite, risk of miscarriage [2]. The demonstration of fetal DNA in maternal plasma in 1997 has opened up possibilities for noninvasive prenatal diagnosis (NIPD) [3].

Maternal plasma DNA contains a mixture of fragmented maternal and fetal genomic DNA [4]. The large background of maternal DNA represents a challenge for the interrogation of fetal chromosomal status. Early studies for NIPD of T21 were polymorphism-based, requiring the measurements of allelic ratios and comparing them with the expected normal values [5]–[7]. These early methods were based on fetal-specific molecular signatures such as DNA methylation markers [5] and RNA markers [6], or required one to attempt to increase the fractional fetal DNA concentration to a sufficiently high level such as using formaldehyde treatment of maternal plasma [7]. However, for the last approach, there are controversies on the effectiveness of formaldehyde treatment because this method could not be replicated consistently by different groups [8]–[10]. Therefore, the clinical applicability of such a method remains unclear.

An alternative approach for NIPD of T21 is to measure the proportion of chromosome 21 (chr21)-derived DNA molecules in a maternal plasma sample. If a mother is carrying a T21 fetus, the additional copy of chr21 from the fetus would contribute an additional amount of chr21 DNA molecules to the maternal plasma sample, leading to an increased proportion of chr21 sequences [11]. The advent of massively parallel sequencing (MPS) enhances the precision of DNA quantification to an unprecedented level, and has enabled the detection of aberrant quantities of fetal DNA derived from an aneuploid chromosome using a tag-counting approach [11]–[14]. Therefore, it is an opportune time to reinvestigate the feasibility of the allelic ratio approach for the NIPD of T21 by using MPS. Since single nucleotide polymorphisms (SNPs) only account for approximately 1.6% of the human genome according to the dbSNP Build 135 for human (http://www.ncbi.nlm.nih.gov/projects/SNP/), conventional non-targeted MPS would only include SNP alleles in a small proportion of sequence reads. In our previous publication, by using a hybridization-based targeted MPS platform, we demonstrated the enrichment of DNA molecules within the targeted regions, as well as the preservation of the allelic ratios of the targeted SNPs in maternal plasma after target enrichment [15]. Therefore, in this study, we investigated the feasibility of the allelic ratio approach by using targeted MPS to preferentially sequence selected SNP loci in maternal plasma DNA for fetal T21 detection.

## Materials and Methods

### Ethics statement

Approvals were obtained from the institutional review board of the Joint Chinese University of Hong Kong - New Territories East Cluster Clinical Research Ethics Committee. All participants gave informed written consent.

### Subjects and sample collection

We recruited fourteen pregnant women with singleton fetuses (gestational age ranging from 12 weeks to 13 weeks and 5 days) from the Department of Obstetrics and Gynaecology, Prince of Wales Hospital, Hong Kong. Maternal peripheral blood samples were collected prior to CVS. Following CVS, fetal genomic DNA samples were obtained from the chorionic villi. The aneuploidy or euploidy status were confirmed by full karyotyping. Among the fourteen cases, seven were T21 fetuses and the rest were euploid.

### DNA extraction

Maternal peripheral blood samples (5–10 mL) were centrifuged at 1 600 g for 10 min at 4°C. The plasma portion (2–5 mL) was recentrifuged at 16 000 g for 10 min at 4°C. We removed any residual plasma from the blood cell portion by recentrifugation at 2 500 g for 5 min [16]. Plasma DNA was extracted with the DSP DNA Blood Mini Kit (Qiagen), as described previously [11]. Fetal and maternal genomic DNA was extracted from chorionic villi and peripheral blood cells, respectively, with the QIAamp DNA Blood Mini Kit (Qiagen) according to the manufacturer's protocol. The extracted plasma DNA was quantified by real-time PCR using an ABI 7300 Sequence Detector (Applied Biosystems). A β-globin real-time PCR assay was performed as described previously [4]. A conversion factor of 6.6 pg of DNA per cell was used to calculate the amount of the extracted plasma DNA.

### Targeted MPS of plasma DNA libraries

As the plasma DNA molecules were already fragmented in nature, no additional fragmentation step was required. 10–30 ng plasma DNA for each case was used for DNA library construction by the Genomic DNA Sample Preparation Kit (Illumina) as previously described [15], except for the replacement of the adaptors and primers with the oligonucleotides from the Multiplexing Sample Preparation Oligonucleotide Kit (Illumina) and the SureSelect Target Enrichment System Kit (Agilent) according to the manufacturer's instructions. In order to obtain high fold sequencing coverage for SNPs, the SureSelect Target Enrichment System (Agilent) was applied for capturing SNPs in the targeted regions. As a proof-of-principle study, approximately 5% of the probes were designed to target a total of 2 906 SNP loci on chr7 (313 SNPs), chr13 (564 SNPs), chr18 (592 SNPs) and chr21 (1 437 SNPs), while the remaining probes were designed for another project. The above 2 906 SNPs represented a subset of the SNPs in the Genome-Wide Human SNP Array 6.0 (Affymetrix) (Table S2). 500 ng of each constructed plasma DNA library was incubated with the probes for 24 h at 65°C. After hybridization, the captured DNA molecules were eluted and amplified by a 12-cycle PCR according to manufacturer's instructions. Libraries with and without target enrichment were indexed for multiplex sequencing on a Genome Analyzer IIx (Illumina) in 50-bp×2 paired-end format. An additional 7 cycles of sequencing were performed to decode the index sequence. All sequenced reads were aligned to the unmasked human reference genome (Hg18) (http://genome.ucsc.edu) with the aid of SOAPaligner/soap2 (http://soap.genomics.org.cn). Two mismatches were allowed during alignment. The range of fragment sizes of paired-end reads was defined as 50–600 bp. Duplicated paired-end reads (e.g., reads with identical sequences and start–end coordinates) were considered clones of the same original plasma DNA template. All but one of the duplicated reads were filtered, leaving only 1 copy for subsequent bioinformatics analysis as previously described [15]. The allele counting in plasma DNA was based on the SNP loci contained in the microarray. Since we already knew the position for each SNP locus, we directly counted the alleles in plasma DNA according to the maternal and fetal genotyping information which served as the gold standard. For example, if maternal genotype was AA and fetal genotype was AB for one SNP, we counted the copy number of A and B alleles for this SNP locus in plasma DNA for subsequent analysis.

### F-S ratio calculation

**Figure 1:**
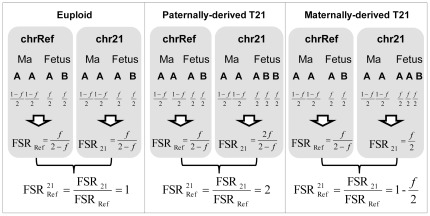
Schematic of T21 detection by F-S ratio calculation. Assuming the fractional fetal DNA concentration in chrRef is f, the F-S ratio would be f/(2-f) on chrRef irrespective of the aneuploidy status of the fetus. On the other hand, the F-S ratio on chr21 would be f/(2-f) if the mother is carrying a euploid fetus, 2f/(2-f) if the mother is carrying a paternally-derived T21 fetus, and f/2 if the mother is carrying a maternally-derived T21 fetus. Therefore, the 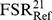 would be 1 if the mother is carrying a euploid fetus, would become 2 if the mother is carrying a paternally-derived T21 fetus, and would become (1-f/2) if the mother is carrying a maternally-derived T21 fetus.

We define a SNP as informative if it is homozygous in the mother (e.g., genotype AA) and heterozygous in the fetus (e.g., genotype AB). In this scenario, the B allele is the fetus-specific allele and the A allele is the allele shared by the mother and fetus. Informative SNPs were identified according to maternal and fetal genotyping information. The fetus-specific allelic counts and shared allelic counts for the informative SNPs were obtained from the plasma DNA sequencing data, and were used to calculate the ratio between the fetus-specific alleles and the shared alleles (expressed as F-S ratio) for chr21 (expressed as FSR_21_) and the reference chromosomes (expressed as FSR_Ref_). In this study, reference chromosomes included all autosomes except chr21 in the non-targeted sequencing data analysis, and included chr7, chr13 and chr18 in the targeted sequencing data analysis. If a mother is carrying a euploid fetus, the ratio between FSR_21_ and FSR_Ref_ (expressed as 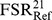) should be equal to 1 (Figure 1). If a mother is carrying a T21 fetus with an additional chr21 from the father (referred in this manuscript as paternally-derived T21), the fetal genotype on chr21 would become ABB. The additional copy of the fetus-specific allele would increase the 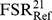 to 2 (Figure 1). If the extra copy of chr21 is from the mother (referred in this manuscript as maternally-derived T21), the fetal genotype on chr21 would become AAB. The additional copy of the shared allele would reduce the 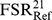 to less than 1 (Figure 1).

### Microarray genotyping

Maternal and fetal genomic DNA samples were genotyped with the Genome-Wide Human SNP Array 6.0 (Affymetrix). The parental origin of the additional copy of chr21 in seven T21 fetuses was determined using microarray analysis of the chorionic villus samples in which the allelic signal intensities for SNPs on chr21 were analyzed. If the fetus has paternally-derived T21, the signal intensity of the fetus-specific allele (B allele) should be 2-fold higher than the shared allele (A allele), because the fetal genotype would become ABB on chr21, and vice versa. Using this approach, one fetus was identified as a paternally-derived T21, while six were maternally-derived T21.

### Computer simulation

Computer simulation was employed to investigate the accuracy of the F-S ratio analysis for T21 detection. The statistical model is based on the assumption that the numbers of fetus-specific and shared allelic counts should follow a binomial distribution, according to the fractional fetal DNA concentration in both the paternally- and maternally-derived T21 models. For example, assuming the fractional fetal DNA concentration in the reference chromosomes (chrRef) is f, the probability of detecting the fetus-specific allele for an informative SNP would be f/2 on chrRef irrespective of the aneuploidy status of the fetus. On the other hand, the probability of detecting the fetus-specific allele on chr21 would be f/2 if the mother is carrying a euploid fetus, 2f/(2+f) if the mother is carrying a paternally-derived T21 fetus, and f/(2+f) if the mother is carrying a maternally-derived T21 fetus (Figure 1). For illustration purposes, we assumed equal amounts of informative allelic counts (the summation of fetus-specific and shared allelic counts) were obtained from chr21 and chrRef. Based on the above assumptions, 1 000 euploid and 1 000 T21 cases were simulated each time for different fractional fetal DNA concentrations to investigate the detection accuracy in both the paternally- and maternally-derived T21 models.

## Results

### Efficiency of target enrichment

We defined sequencing depth as the mean number of times each base had been sequenced in a particular region. On average, 3.1 million and 2.9 million paired-end reads in non-enriched and enriched samples, respectively, were mapped uniquely to the reference human genome (Hg18). After filtration of the duplicated paired-end reads, we calculated the sequencing depth of the targeted region by dividing the total number of sequenced bases within the targeted region by the length of the targeted region. The mean sequencing depth was 0.12 time for the non-enriched samples and 27 times for the enriched samples, indicating a mean enrichment of 225-fold (Table S1). This finding was consistent with our previous publication [15].

### Estimation of fractional fetal DNA concentrations

We calculated the fractional fetal DNA concentrations (also referred to as the fetal DNA proportion) in the non-enriched samples by using the informative SNPs from all autosomes except for chr21, according to the equation: fractional fetal DNA concentration=fetus-specific allelic counts ×2/(fetus-specific allelic counts + shared allelic counts) ×100%. The median fractional fetal DNA concentration for the fourteen cases analyzed in this study was 15.5% (range: 9.1%–19.7%).

### F-S ratio calculation using non-targeted sequencing data

**Figure 2:**
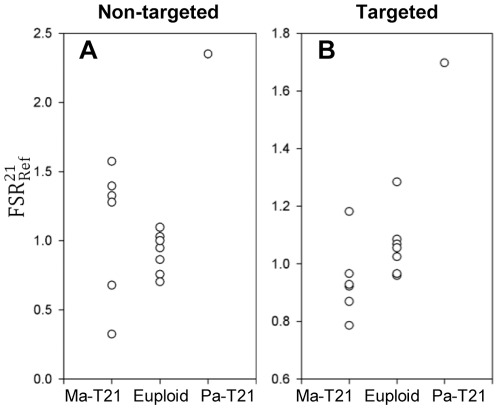
T21 detection by F-S ratio in non-targeted and targeted sequencing data. 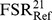
 values were calculated to differentiate the paternally- and maternally-derived T21 from the euploid fetuses in non-targeted (A) and targeted (B) sequencing data. (Ma-T21: maternally-derived T21. Pa-T21: paternally-derived T21.).

Based on maternal and fetal genotyping information, informative SNPs were identified on chr21 (range amongst the samples: 1 044 to 1 775 SNPs), and chrRef which included all autosomes except chr21 (range amongst the samples: 99 581 to 106 950 SNPs). Within the above informative SNPs, fetus-specific allelic counts (expressed as FC) and shared allelic counts (expressed as SC) were determined for chr21 (FC_21_=2 to 19, SC_21_=87 to 213) and chrRef (FC_Ref_=390 to 1 622, SC_Ref_=6 880 to 18 892). We then calculated the FSR_21_, FSR_Ref_ and 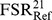 (Table S1). As shown in Figure 2A, the paternally-derived T21 case (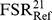
=2.35) could be differentiated from the euploid group (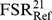 mean 0.91, median 0.96, range: 0.70 to 1.10). On the other hand, the maternally-derived T21 cases (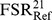 mean 1.10, median 1.30, range: 0.33 to 1.57) overlapped with the euploid cases (Mann-Whitney rank sum test, p=0.366).

### F-S ratio calculation using targeted sequencing data

Among the 1 437 targeted SNP loci on chr21 and 1 469 SNP loci on chrRef (including chr7, chr13 and chr18), informative SNPs (chr21=151 to 273 SNPs, chrRef=145 to 182 SNPs) were identified for each case. The number of sequenced reads with the fetus-specific and shared alleles were determined for chr21 (FC_21_=197 to 761, SC_21_=3 610 to 7 740) and chrRef (FC_Ref_=154 to 473, SC_Ref_=2 786 to 5 557) (Table S1). As shown in Figure 2B, the paternally-derived T21 case (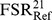
=1.68) could be differentiated from the euploid group (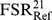 mean 1.06, median 1.06, range: 0.95 to 1.29). Although the maternally-derived T21 group appeared to have lower 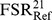 values (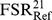 mean 0.94, median 0.93, range: 0.78 to 1.17) (Mann-Whitney rank sum test, p=0.051), there was still significant overlapping with the euploid group.

### Computer simulation

**Figure 3:**
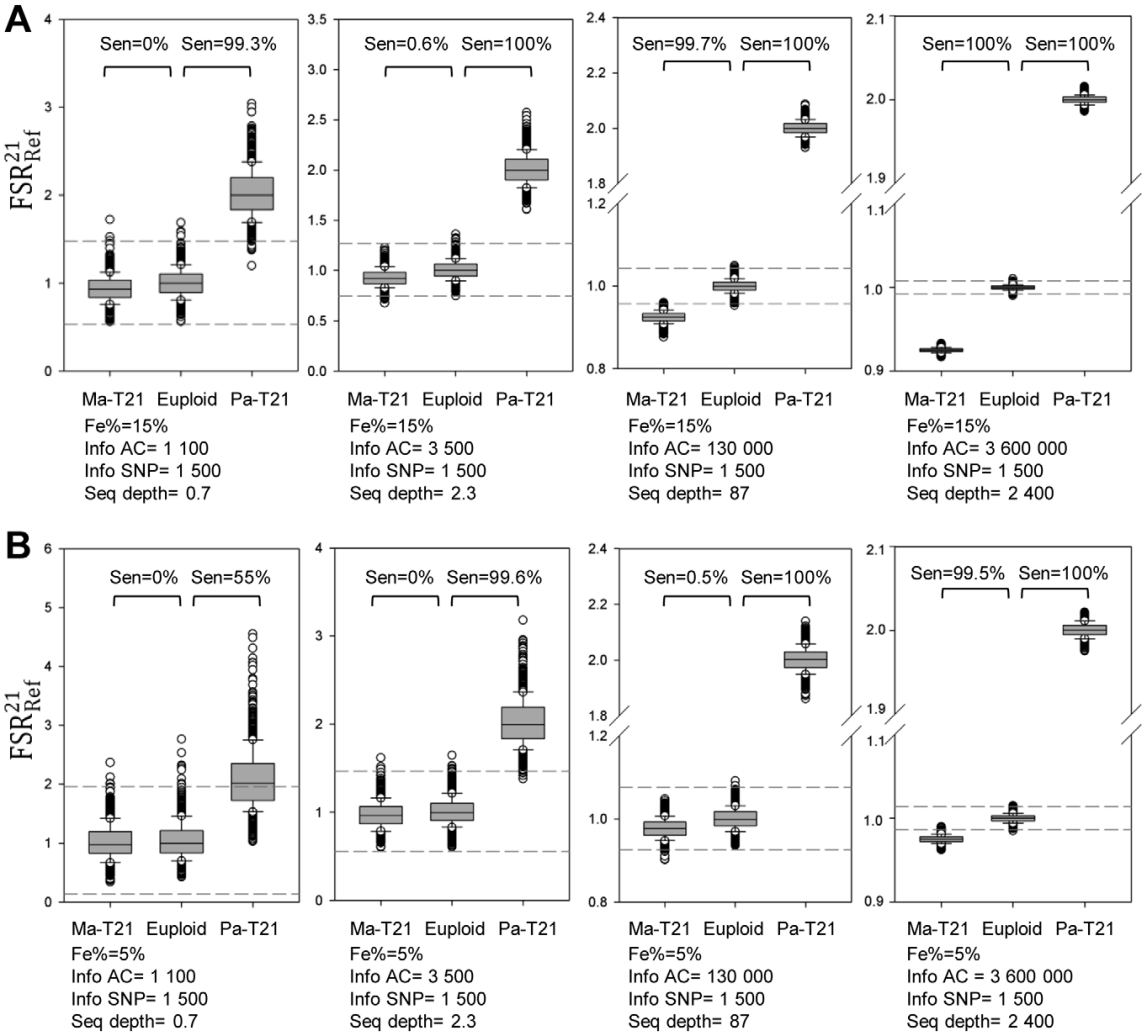
Computer simulation for T21 detection for fractional fetal DNA concentrations of 5% and 15%. In order to obtain a specificity of greater than 99%, the cutoffs for T21 differentiation were chosen at 3 standard deviations above and below the mean F-S ratio of the euploid group. The sensitivity for paternally- and maternally-derived T21 detection was investigated on different numbers of informative allelic counts on chr21 and chrRef, respectively, for a fractional fetal DNA concentration of 15% (A). Similar analysis was performed for a fractional fetal DNA concentration of 5% (B). (Ma-T21: maternally-derived T21. Pa-T21: paternally-derived T21. Sen=sensitivity. Fe%=fractional fetal DNA concentration. Info AC=informative allelic counts on each of chr21 and chrRef. Info SNP=informative SNPs on each of chr21 and chrRef. Seq depth=sequencing depth. Info AC=Info SNP×Seq depth).

**Figure 4:**
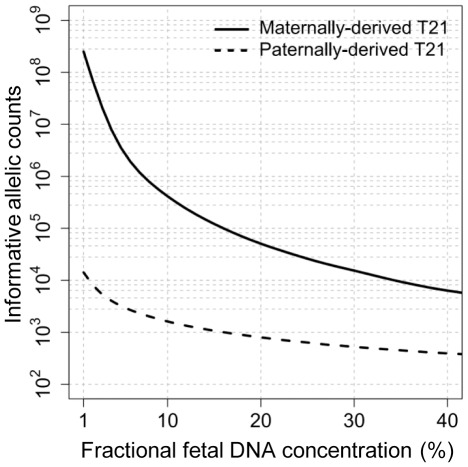
Computer simulation to investigate the minimal number of informative allelic counts for T21 detection. The solid curve represents the minimal number of informative allelic counts required on each of chr21 and chrRef (Y axis), in order to achieve a reliable detection in maternally-derived T21 (sensitivity >99%, specificity >99%) according to a given fractional fetal DNA concentration (X axis). The dash curve represents the paternally-derived T21 model.

We used computer simulation to determine the parameters that would further improve the accuracy of the allelic ratio approach for T21 detection. First, we fixed the fractional fetal DNA concentration at 15% and gradually increased the numbers of informative allelic counts on chr21 and chrRef to investigate the detection accuracy for fetal T21. Additional informative allelic counts would improve the detection accuracy in both the paternally- and maternally-derived T21 models. In order to obtain accurate detection (sensitivity >99%, specificity >99%), more informative allelic counts were required for the maternally-derived T21 detection (informative allelic counts=130 000) than the paternally-derived T21 detection (informative allelic counts=1 100) (Figure 3A). If the fractional fetal DNA concentration was reduced to 5%, the corresponding informative allelic counts would need to be increased to 3 600 000 for detecting maternally-derived T21 and 3 500 for detecting paternally-derived T21 (Figure 3B). When the fractional fetal DNA concentration in maternal plasma was gradually decreased from 40% to 1%, the minimal number of informative allelic counts would need to be increased in both the paternally- and maternally-derived T21 scenarios, in order to maintain a high detection rate (sensitivity >99%, specificity >99%). The required increase was higher for the maternally-derived T21 scenario than that for the paternally-derived one (Figure 4). The sensitivity and specificity chosen for this analysis were chosen to mirror the recently reported performance of the non-polymorphic tag counting approach [12]–[14].

## Discussion

In this study, we investigated if the analysis of chromosome-specific allelic ratio would be useful for the NIPD of fetal chromosomal aneuploidy. The paternally-derived T21 case was successfully detected in both non-targeted and targeted sequencing data. For the maternally-derived T21 cases, this approach became less effective. Although the mean 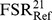 values was lower in the maternally-derived T21 cases, there was significant overlapping in the 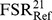 values between the euploid and the maternally-derived T21 cases. The difference in performances between paternally- and maternally-derived T21 detection was mainly due to the magnitude of the change in 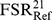, which was increased by 2-fold for paternally-derived T21 and decreased by f/2-fold for maternally-derived T21 (Figure 1). Since fetal DNA represents a minor population in maternal plasma, the lower fractional concentration of fetal DNA would diminish the degree of 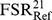 change in maternally-derived T21. For example, assuming the fractional fetal DNA concentration is 5%, the 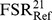 of maternally-derived T21 would become 0.975, which is very close to that of a euploid case (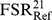
=1).

Since maternally-derived T21 accounts for the majority of T21 cases in clinical practice, it is necessary to investigate how one could improve its detection accuracy using the allelic ratio method [1], [17]. One approach is to increase the fractional concentration of fetal DNA, which would enlarge the magnitude of 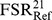 change in maternally-derived T21. Although an early study attempted to enrich the fetal DNA proportion by formaldehyde treatment of maternal plasma [7], this method is not ready for use because it has not been consistently reproduced by different groups [8]–[10]. Alternatively, the accuracy of detecting maternally-derived T21 can be improved by increasing the number of informative allelic counts. According to our simulation analysis, additional informative allelic counts would, to some extent, compensate for the loss of detection accuracy caused by the decrease in fractional fetal DNA concentration (Figure 4). Two approaches can be used to increase the number of informative allelic counts, namely, recruiting more informative SNPs and sequencing each locus deeper.

The number of informative SNPs is determined by two factors: the total number of SNPs on the chromosome of interest for detection and the frequency of informative SNPs. The latter is the percentage of detectable informative SNPs amongst a group of analyzed SNPs. As a proof-of-principle study, we directly used the fetal genotype information, as determined by microarray-based analysis of chorionic villus DNA, to maximize the frequency of informative SNPs. Amongst the fourteen cases recruited for this study, the mean proportions of SNPs that were informative were 11.9% (range: 11.3% to 12.1%) in all autosomes and 12.9% (range: 11.0% to 15.0%) in targeted SNP loci. However, the fetal genomic material would not be available beforehand for actual NIPD. Therefore, informative SNPs would need to be deduced indirectly, for example, by selecting SNPs in which the parents are both homozygous but with different alleles (e.g., mother is AA and father is BB). In this scenario, the frequency of informative SNPs would decrease, because such an approach would exclude any SNPs in which the father is heterozygous.

In terms of targeted analysis of SNPs, we demonstrated in this study the feasibility of the hybridization-based enrichment system. In this proof-of-principle study, we only employed a relatively small number of probes to capture 2 906 SNP loci on the target chromosomes. However, it is possible to increase the number of analyzed SNP loci. For example, if we design probes to cover all chr21 SNP loci (12 930 SNPs) on the Genome-Wide Human SNP Array 6.0 (Affymetrix), we could increase the number of informative SNPs on chr21 to approximately 1 500 SNPs for each case in the current dataset. If we obtain the same number of informative SNPs on chrRef and sequence the plasma DNA to a depth of 87 times, we would harvest approximately 130 000 informative allelic counts (1 500×87) on chr21 and chrRef, respectively. Such numbers would allow the relatively robust classification of maternally-inherited T21 from the euploid cases, assuming a fractional fetal DNA concentration of 15% or above (Figure 3A). Given that in this study the mean frequency of informative SNPs is approximately 12% and approximately 50% reads could be mapped back to the targeted regions (with the remaining reads being off-target), the total number of reads required to obtain 130 000 informative allelic counts would be approximately 4.3 million (130 000 reads ×2/(12%×50%)), which was comparable to that of the tag counting approach [12]–[14]. However, if the fractional fetal DNA concentration is reduced to 5%, the number of reads would need to be increased to approximately 120 million (Figure 3B), which was more than that needed for the non-polymorphic tag counting approach [12]–[14]. 5% and 15% were analyzed here because 5% is close to the lower limit for the non-polymorphism-based tag counting approach at the depth of sequencing used in a number of recent studies, and 15% is the approximate mean fractional fetal DNA concentration in the published clinical trials based on the tag counting approach [12]–[14].

In order to obtain a sufficient number of allelic counts, an alternative approach is to sequence more deeply each locus with a relatively small number of informative SNPs. According to our previous publication, we demonstrated that each milliliter of plasma contained approximately 1 000 genome-equivalents of DNA, which would give rise to 2 000 allelic counts for each locus [4]. If we could count all of the alleles for each locus in 5 mL plasma, we would obtain 10 000 allelic counts for each locus. In this scenario, 26 informative SNPs (13 SNPs on chr21, 13 SNPs on chrRef) could provide sufficient informative allelic counts for maternally-derived T21 detection in a sample containing 15% fetal DNA, representing 130 000 informative allelic counts (10 000×13) on chr21 and chrRef, respectively. The number of informative SNPs would need to be increased to 720 (360 SNPs on chr21, 360 SNPs on chrRef) for a sample containing a fractional fetal DNA concentration of 5%. Due to the high read depth required by this strategy (i.e. 10 000 counts per informative allele), PCR-based approaches for target enrichment might be alternative methods for this type of application [18], [19].

The targeted massively parallel sequencing of SNP loci in plasma DNA is thus a feasible method for the NIPD of fetal aneuploidies. However, when compared with published work based on tag counting of nonpolymoprhic loci for T21 detection [12]–[14], this approach appears to be less effective and robust because it requires the parental genotyping information and needs more sequencing reads if the fractional fetal DNA concentration is low as illustrated in this proof-of-principle study. In addition, the costs and time required for target enrichment would need to be taken into account. Nevertheless, this study has also outlined the strategies that one could use to further improve this approach.

## Supporting Information

Table S1 **Sequencing data and allelic information for plasma DNA libraries with and without target enrichment.**
(DOC)

Table S2 **Information of SNPs used for targeted sequencing.**
(XLS)
