# Supplementary material for: Noninvasive Prenatal Diagnosis of Fetal Trisomy 21 by Allelic Ratio Analysis Using Targeted Massively Parallel Sequencing of Maternal Plasma DNA
Source: PLoS One. 2012 May 29;7(5):e38154. doi: 10.1371/journal.pone.0038154 (PMC3362548; doi:10.1371/journal.pone.0038154)
Supplement: Table S1 — Sequencing data and allelic information for plasma DNA libraries with and without target enrichment. (DOC) [file pone.0038154.s001.doc]

**Table S1. Sequencing data and allelic information for plasma DNA libraries with and without target enrichment.**

| No. | Sample name | Fetal status | Target enriched | Mapped  reads^a^ | Target  seq depth | chr21 | | | | chrRef | | | | $\text{FSR}_{\text{Ref}}^{21}$ |
| --- | --- | --- | --- | --- | --- | --- | --- | --- | --- | --- | --- | --- | --- | --- |
|  |  |  |  |  |  | Info SNP | SC | FC | FSR | Info SNP | SC | FC | FSR |  |
| 1 | PW257 | euploid | No | 2 M | 0.07 | 1674 | 110 | 8 | 0.073 | 103563 | 6880 | 662 | 0.096 | 0.76 |
|  |  |  | Yes | 3.3 M | 31.18 | 268 | 7559 | 739 | 0.098 | 169 | 4951 | 377 | 0.076 | 1.29 |
| 2 | PW279 | euploid | No | 2.4 M | 0.09 | 1686 | 117 | 9 | 0.077 | 101227 | 7954 | 871 | 0.11 | 0.7 |
|  |  |  | Yes | 3.1 M | 30.3 | 269 | 7296 | 761 | 0.104 | 148 | 4129 | 449 | 0.109 | 0.95 |
| 3 | PW280 | euploid | No | 2.8 M | 0.11 | 1373 | 141 | 9 | 0.064 | 103732 | 10423 | 703 | 0.067 | 0.96 |
|  |  |  | Yes | 2.8 M | 24 | 186 | 3968 | 222 | 0.056 | 162 | 3934 | 228 | 0.058 | 0.97 |
| 4 | PW338 | euploid | No | 2.3 M | 0.09 | 1364 | 121 | 5 | 0.041 | 99581 | 8136 | 390 | 0.048 | 0.85 |
|  |  |  | Yes | 3.2 M | 28.39 | 221 | 6164 | 285 | 0.046 | 162 | 4245 | 181 | 0.043 | 1.07 |
| 5 | PW263 | euploid | No | 3 M | 0.11 | 1592 | 162 | 14 | 0.086 | 105140 | 10821 | 908 | 0.084 | 1.02 |
|  |  |  | Yes | 2.5 M | 23.35 | 253 | 5539 | 453 | 0.082 | 178 | 3770 | 301 | 0.08 | 1.03 |
| 6 | PW295 | euploid | No | 3.3 M | 0.12 | 1775 | 203 | 17 | 0.084 | 105072 | 11742 | 983 | 0.084 | 1 |
|  |  |  | Yes | 3.1 M | 30.03 | 273 | 7740 | 695 | 0.09 | 156 | 4103 | 345 | 0.084 | 1.07 |
| 7 | PW305 | euploid | No | 3.5 M | 0.13 | 1436 | 167 | 18 | 0.108 | 106341 | 12711 | 1250 | 0.098 | 1.1 |
|  |  |  | Yes | 2.4 M | 21.95 | 191 | 3610 | 343 | 0.095 | 181 | 3866 | 348 | 0.09 | 1.06 |
| 8 | PW146 | T21 | No | 2.6 M | 0.1 | 1173 | 107 | 2 | 0.019 | 106925 | 9703 | 560 | 0.058 | 0.33 |
|  |  |  | Yes | 2.1 M | 21.43 | 213 | 4536 | 197 | 0.043 | 145 | 2786 | 154 | 0.055 | 0.78 |
| 9 | PW150 | T21 | No | 2.7 M | 0.1 | 1044 | 87 | 10 | 0.115 | 106601 | 10259 | 502 | 0.049 | 2.35 |
|  |  |  | Yes | 2.8 M | 26.01 | 151 | 3878 | 250 | 0.064 | 180 | 4317 | 164 | 0.038 | 1.68 |
| 10 | PW178 | T21 | No | 2.9 M | 0.11 | 1049 | 106 | 18 | 0.17 | 106607 | 10263 | 1108 | 0.108 | 1.57 |
|  |  |  | Yes | 2.5 M | 22.83 | 169 | 3675 | 398 | 0.108 | 182 | 3610 | 331 | 0.092 | 1.17 |
| 11 | PW266 | T21 | No | 2.8 M | 0.11 | 1193 | 123 | 17 | 0.138 | 104560 | 10002 | 991 | 0.099 | 1.39 |
|  |  |  | Yes | 3 M | 29.07 | 165 | 4464 | 375 | 0.084 | 156 | 4095 | 396 | 0.097 | 0.87 |
| 12 | PW352 | T21 | No | 4.6 M | 0.17 | 1116 | 146 | 19 | 0.13 | 105157 | 16505 | 1622 | 0.098 | 1.33 |
|  |  |  | Yes | 3.5 M | 30.65 | 181 | 5153 | 483 | 0.094 | 170 | 4649 | 473 | 0.102 | 0.92 |
| 13 | PW392 | T21 | No | 3.1 M | 0.12 | 1189 | 126 | 10 | 0.079 | 105838 | 11464 | 712 | 0.062 | 1.27 |
|  |  |  | Yes | 3.5 M | 34.31 | 186 | 5934 | 325 | 0.055 | 168 | 5557 | 328 | 0.059 | 0.93 |
| 14 | PW421 | T21 | No | 5.2 M | 0.19 | 1206 | 213 | 12 | 0.056 | 106950 | 18892 | 1570 | 0.083 | 0.68 |
|  |  |  | Yes | 3.2 M | 25.45 | 192 | 4397 | 306 | 0.07 | 171 | 4231 | 305 | 0.072 | 0.97 |

^a^ Mapped reads represent sequence reads (read 1 and read 2) both mapping uniquely to the unmasked Hg18 reference genome by SOAP2 alignment, allowing 2 mismatches.

Target seq depth: sequencing depth of the targeted region

Info SNP: informative SNP.

SC: shared allelic counts.

FC: fetus-specific allelic counts.

FSR: F-S ratio, ratio between the fetus-specific allele and the shared allele.

$\text{FSR}_{\text{Ref}}^{21}$= FSR_21_: FSR_Ref_

chrRef: reference chromosome.

T21: trisomy 21.

M: million
